# Contribution of daily and seasonal biorhythms to obesity in humans

**DOI:** 10.1007/s00484-014-0871-z

**Published:** 2014-07-18

**Authors:** Dominika Kanikowska, Maki Sato, Janusz Witowski

**Affiliations:** 1 Department of Pathophysiology, Poznań University of Medical Sciences, Rokietnicka 8, 60-806 Poznań, Poland; 2 Department of Physiology, School of Medicine, Aichi Medical University, Aichi, Japan

**Keywords:** Adipose tissue, Biological clocks, Biometeorology, Circadian rhythm, Food intake, Metabolism, Obesity, Seasonal rhythms, Sleep, Shift work

## Abstract

While the significance of obesity as a serious health problem is well recognized, little is known about whether and how biometerological factors and biorhythms causally contribute to obesity. Obesity is often associated with altered seasonal and daily rhythmicity in food intake, metabolism and adipose tissue function. Environmental stimuli affect both seasonal and daily rhythms, and the latter are under additional control of internal molecular oscillators, or body clocks. Modifications of clock genes in animals and changes to normal daily rhythms in humans (as in shift work and sleep deprivation) result in metabolic dysregulation that favours weight gain. Here, we briefly review the potential links between biorhythms and obesity in humans.

By affecting approximately 400 million people worldwide and with its increasing prevalence, obesity is a significant global health problem (Finucane et al. 2011). It is a well-recognized risk factor for metabolic and cardiovascular disease (Klein et al. 2004). This impact is related partly to altered adipose tissue metabolism and chronic low-grade inflammation (Lumeng and Saltiel 2011).

## Seasonal and daily influence on metabolism and body weight

The amount of body fat may significantly change over the seasons, particularly in latitudes away from the equator, where seasonal changes in climate, temperature and duration of daylight are greater. These lead to changes in availability of certain foods and in individuals’ feeding habits and outdoor activity (resulting e.g. in picnics being more common in summer) (Reilly and Peiser 2006). Plasqui and Westerterp (2004) have shown that there is a significant seasonal variation in physical activity and total energy expenditure, with lower amounts in winter, in young Dutch adults. The biological significance of such seasonal and daily environmental rhythms has long been appreciated (Reinberg 1972) and is best seen in seasonal animals and hibernators, which adjust their physiology both in preparation for, and in response to, changing demands of the environment (Ebling and Barrett 2008). However, in humans living in modern societies, the impact of seasonality has somewhat diminished following the introduction of artificial lighting and heating and air-conditioning systems. The use of these artificial aids reduces the exposure of individuals to fluctuations in ambient temperature and light, and this is more convenient for practising a modern lifestyle. However, these natural fluctuations contribute to the normal adjustment of the body clock to a 24-h period; their extensive use (artificial aids) will lessen this synchronization and may increase the risk of developing *mismatches* between the natural environment and the body clock (similar to the problems observed after a time-zone transition or during night work). It has been claimed that these misalignments may lead to alterations in metabolism and thermoregulation that promote obesity (Johnson et al. 2011; Wyse et al. 2011).

While seasonal rhythmicity in energy storage and expenditure is significantly influenced by changes in the external environment (Reilly and Peiser 2006), the nature of daily rhythms in metabolism is more complex. In this respect, humans possess internal timing mechanisms which can act independently of daily changes in the environment. All cells show a genetic potential for daily rhythmicity, but in practice, this rhythmicity is manifested in only some regions of the body. These regions include the liver (which possesses a food-entrainable oscillator) and the suprachiasmatic nucleus (SCN) paired structures in the base of the hypothalamus. The SCN normally coordinates rhythmic activity throughout the body (acting via the autonomic nervous system, temperature regulation, hormone secretion, sleep, and feeding behaviour) and is known as the “body clock.” Evidence is accumulating to suggest that the disruption of these body clocks may contribute to metabolic disorders and predispose to obesity (Eckel-Mahan and Sassone-Corsi 2013).

## Basic principles of chronobiology

Rhythmicity seen in many processes, including metabolism, reflects both personal habits (e.g. sleep, activity and mealtimes) and the impact of internal body clocks. Humans, like other organisms, possess a timing system that consists of self-sustained oscillators that are reset by various synchronizers. In the absence of time cues, the dominant component of this system free runs with a period of 24–25 h, giving rise to a so-called *circadian* rhythm (from the Latin: *circa diem*—about a day). Normally, this rhythm is entrained or synchronized to a 24-h cycle (called *daily* in this review), predominantly by natural light–dark cycles and, to lesser extent, by cycles of rest and activity or feeding and fasting. External stimuli that can synchronize the body clock to a 24-h cycle are called *zeitgebers* (from German: time givers) (Reilly and Peiser 2006).

The molecular mechanisms that underlie the function of cellular clocks are the oscillating post-translational modifications of proteins (e.g. phosphorylation) and the autoregulatory feedback loops that control gene transcription and translation. The main loop comprises a positive and a negative limb that are interconnected (Albrecht 2012). It consists of the transcriptional activators CLOCK and BMAL1 and their target genes *Per* (period) and *Cry* (cryptochrome). Products of these genes accumulate gradually and inhibit CLOCK-BMAL1 transcription. In turn, the feedback loop controlling BMAL1 involves nuclear receptors REV-ERBα and RORα/β that inhibit and activate BMAL1 transcription, respectively. In addition, the activity of REV-ERBα links metabolism to the clock system (Liu et al. 2007). Administration of REV-ERB ligands in mice has been found to alter expression of both the clock genes in the hypothalamus and the metabolic genes in the liver, skeletal muscles and adipose tissue, resulting in increased energy expenditure (Solt et al. 2012). For details, the reader is referred to recent excellent reviews (Albrecht 2012; Bass 2012; Bass and Takahashi 2010; Mohawk et al. 2012).

## Peripheral and central oscillators

The observation that all cells, tissues and organs contain the molecular potential to manifest a clock gave rise to the concept of *peripheral* and *central* (master) oscillators, the former normally being subservient to the latter. The central pacemaker in mammals is located in two hypothalamic clusters of neurons, the SCN. These centres control behavioural, metabolic and physiological rhythms and can synchronize the peripheral oscillators (Welsh et al. 2010). An important peripheral oscillator is the food-entrainable oscillator (FEO), which controls food-anticipatory activity (FAA) in rodents, the exact location of which is unknown (Mieda et al. 2006). FAA in rodents is an increase in activity just before the food is regularly available. Daily rhythms of locomotor activity, body temperature and corticosterone secretion can thus synchronize with the rhythm of food availability (Stephan 2002), even when food is presented during the resting phase or when the central oscillator is destroyed (Mistlberger 2011). These observations indicate that the FEO (and, possibly, peripheral clocks in general) can act independently of the SCN, at least in some animal. This independence of activity may become important when the master oscillator and lifestyle become misaligned.

In normal circumstances, the master clock, the environment and peripheral clocks are synchronized to one another (Reilly and Peiser 2006).

Whereas periodicity of the master clock is controlled mainly through the light–dark cycle (acting as a zeitgeber), peripheral oscillators are affected either by behaviour (cycles of rest and activity or feeding and fasting) or by fluctuations in the levels of circulating hormones, such as catecholamines (Dibner et al. 2010) and glucocorticoids (Balsalobre et al. 2000). For example, peripheral oscillators in the pancreas and the liver (Marcheva et al. 2010) can be adjusted by regular food intake, even if this intake is timed unusually.

## The rhythmic organism

A measured daily rhythm consists of endogenous and exogenous components. The SCN produces the *endogenous* component of the observed rhythm, and the *exogenous* component is superimposed upon it and corresponds to the environment and lifestyle (e.g. inactivity and fasting when asleep). In practice, therefore, rhythms measured in the presence of an exogenous component may not give clear information regarding the activity of the internal oscillators, and the implications of this will be considered at the end of this review. However, normally, the endogenous and exogenous components are in phase but may become desynchronized (e.g. by night work), because the SCN is rather slow to adjust (Reilly and Peiser 2006). Under such circumstances, the independent role of peripheral oscillators becomes important. The environment and lifestyle may also adjust the timing of the master oscillator (e.g. by changing the light–dark cycle) and of the peripheral oscillators (e.g. by changing feeding times, which will impact on FEO).

Food intake in humans shows not only a daily rhythm (daytime rather than nocturnal eating) but also a rhythm related to the intake of individual meals with a period of about 4–5 h. This rhythm is *ultradian*, having a period of oscillation less than 20 h. Such rhythms are probably seen in the activity of all hormones associated with food metabolism. While they are linked directly to food intake (and, as such, can be considered exogenous), other rhythms are likely to be derived from internal oscillators. Episodic release of hormones may exhibit yet another periodicity that lasts for minutes and reflects pulsatile release of a hormone followed by its removal and breakdown. There are also *infradian* rhythms (with periodicity greater than 28 h) including seasonal rhythms. Since there is no clear evidence that endogenous circannual oscillators exists in humans, seasonal rhythms observed are attributed rather to exogenous factors. As indicated earlier, they include seasonal variations in food intake, physical activity, ambient temperature and the duration of daylight. Less information is available regarding seasonal rhythms and obesity in humans. This lack of information is partly due to the obvious fact that such studies demand a more elaborate protocol which covers the four seasons. Moreover, if any interactions between daily rhythms and seasonal rhythms are sought, then the a full set of data covering the 24 h must be collected four times per year.

## Rhythmicity of food intake, gut function and metabolism

### Food intake

Daily food intake in humans usually consists of two to three main meals consumed at times that depend largely on lifestyle and social factors. It has been observed that food intake in the morning is more satiating than the same meal eaten in the evening (de Castro 2009); it has also been observed that the amount of food eaten shows seasonal variations, with increased meal size and total calorie intake occurring in the autumn (de Castro 1991). The hunger and satiety centres in the hypothalamus contain receptors for mediators that affect feeding behaviour. These substances are either orexigenic (stimulate feeding) or anorexigenic (inhibit feeding) (Naslund and Hellstrom 2007). Short-term regulation of food intake involves cholecystokinin (Little et al. 2005), peptide YY, glucagon-like peptide and insulin (Suzuki et al. 2012), all of which act anorexigenically, and ghrelin, which stimulates appetite (Cummings 2006). These mediators display daily and ultradian rhythms in phase with food intake and some of them (ghrelin, leptin, insulin) are also involved in long-term regulation of body weight and energy homeostasis (Stutz et al. 2007).

The mechanisms controlling seasonal food intake and energy balance in seasonal animals and hibernators differ across the species and reflect different strategies employed to survive in a harsh environment (Florant and Healy 2012). The seasonal changes are executed through fluctuations in humoral signals, including leptin and ghrelin (Adam and Mercer 2004; Florant and Healy 2012). Interestingly, while leptin concentrations in humans do not exhibit consistent seasonal changes, it has been observed that the levels of leptin, cholesterol and triglycerides in obese males are significantly higher in winter (Kanikowska et al. 2013).

### Gut function

The digestive system shows rhythmicity in many functions, including basal gastric acid secretion, epithelial cell proliferation and gastrointestinal motility (Ekmekcioglu and Touitou 2011). It is attributed primarily to the timing and the size of meals and exhibits daily and ultradian components. However, some aspects of this rhythmicity may reflect the function of a peripheral clock. For example, it has been demonstrated in animals that rhythmic expression of clock genes within the neurons of the myenteric plexus modulates colonic motility by controlling the expression of neuronal nitric oxide synthetase (nNOS) and vasoactive intestinal peptide (VIP) (Hoogerwerf 2010). Indirect evidence for a role of biological clocks in gastrointestinal functions in humans comes from observations of night workers who have altered appetites and a higher prevalence of constipation, diarrhoea and abdominal discomfort (Nojkov et al. 2010). They are also at increased risk of obesity (Lowden et al. 2010).

### Energy storage and expenditure

Metabolism of absorbed foodstuffs shows rhythmicity that reflects changes in the release of endocrine regulators. Hormones associated with metabolism (including glucagon, insulin, catecholamines, glucocorticoids and thyroid hormones) show both circadian and ultradian oscillations. These rhythms are dominated by food intake (and as such are exogenous and ultradian) but may also be influenced by the SCN that produces daily fluctuations in sympathetic nervous system outflow which may affect hormone secretion (e.g. insulin).

Rhythmicity in clock gene expression and adipocytokine release is seen also in white and brown adipose tissues (WAT and BAT, respectively) (Gavrila et al. 2003). Body temperature in adult humans is about 1 °C higher during the day, which is attributed both to the sleep-wake cycle (exogenous component) and to the SCN-generated rhythmicity in metabolism and peripheral vasculature tone (endogenous component). In babies, however, these rhythms of temperature regulation are not fully developed, and BAT plays an important role in heat production. BAT shows clock gene expression (Zvonic et al. 2006) and shows a 24-h rhythm of glucose uptake (van der Veen et al. 2012). Moreover, BAT activity is regulated via the sympathetic nervous system and by several hormones, all of which express daily variations (Kriegsfeld and Silver 2006). Seasonal changes in BAT have been observed in humans, with BAT growth induced by exposure to cold and associated with the shorter hours of daylight in the autumn and winter (Au-Yong et al. 2009; Saito et al. 2009).

**Fig. 1:**
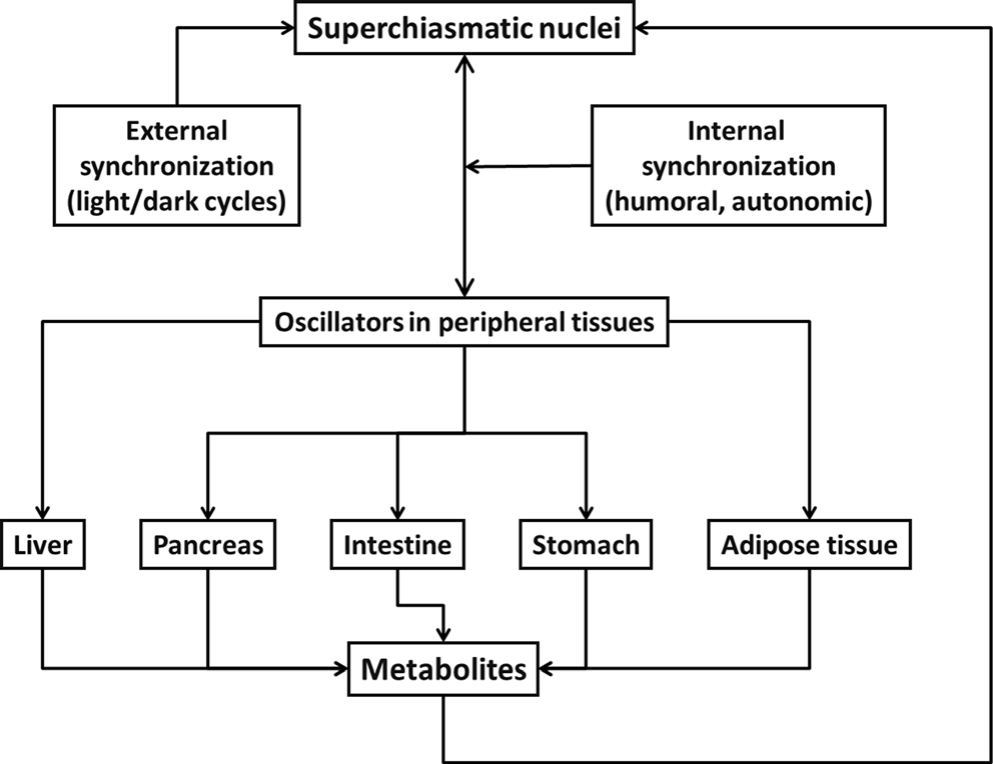
Interactions between the “master” clock (SCN), peripheral oscillators and the environment

It is now clear that several aspects of metabolism show daily rhythmicity related to SCN function (Bass 2012; Bass and Takahashi 2010; Marcheva et al. 2013). However, the SCN is also adjusted by feeding behaviour and metabolic products, which results in additional ultradian rhythmicity. Moreover, many organs involved in food metabolism, such as the liver (Balsalobre et al. 2000), pancreas (Sadacca et al. 2011), intestine and stomach (Bostwick et al. 2010) and the adipose tissue (Johnston 2012), have autonomous peripheral oscillators (Fig. 1). Therefore, it is not surprising that factors altering these interactions might adversely impact on metabolism and be associated with an increased risk of obesity. In this respect, it has recently been shown that the impairment of peripheral clocks may be associated with the development of diabetes (Pappa et al. 2013).

## Factors associated with increased risk of obesity

The obvious causes of obesity include excessive food intake and inadequate physical activity. These topics are extensively covered by many reviews (Boulos et al. 2012). However, other lifestyle-related factors that have been implicated in obesity (such as sleep duration, eating habits, shift work) have not always received enough attention (Chaput et al. 2010).

### Sleep duration

**Fig. 2:**
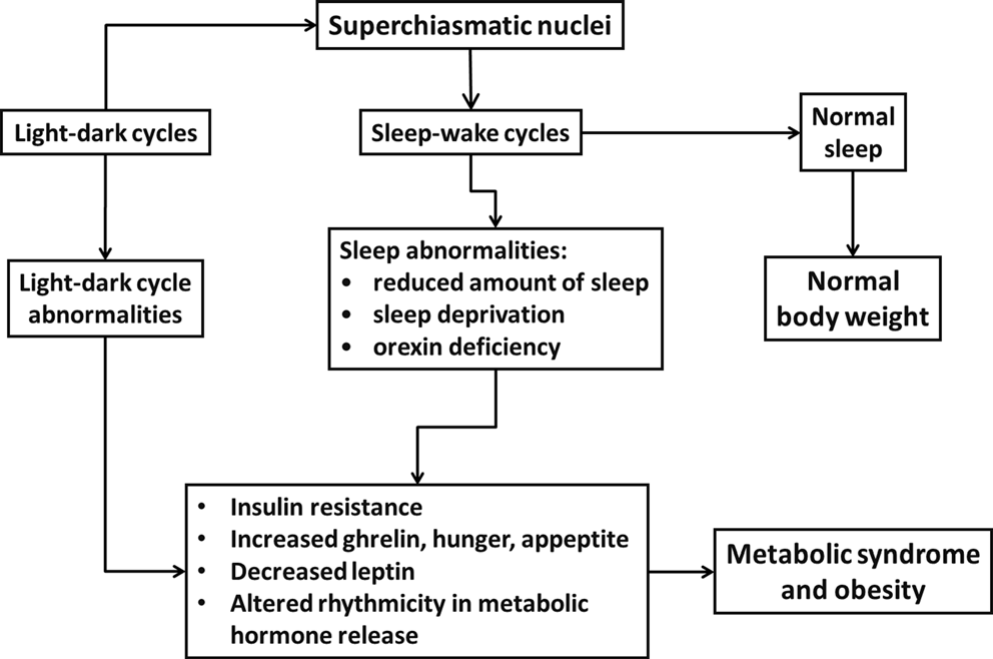
Possible effects of altered sleep-wake cycles on metabolic hormones and body weight

Short sleep (defined as ≤6 h of sleep per day) and sleep disorders have been associated with lower concentrations of leptin and higher levels of ghrelin and with increased hunger and appetite (Taheri et al. 2004). In addition, it has been demonstrated that sleep deprivation may contribute to obesity through modulating plasma levels of leptin (Mullington et al. 2003). Also, the lack of orexin, a hypothalamic wakefulness-inducing neuropeptide (Sakurai et al. 1998), affects sleep, feeding and metabolism (Adamantidis and de Lecea 2008) and is associated with increased likelihood of developing obesity (Funato et al. 2009). Narcolepsy, when patients suffer from extreme daytime sleepiness due to the loss of orexin-producing neurons (Tsujino and Sakurai 2013), has also been linked with increased body mass and obesity (Kotagal et al. 2004). Patients with night eating syndrome, in whom the patterns of sleep and eating are disrupted, are often obese (Colles et al. 2007) and have altered rhythms of plasma leptin, insulin, cortisol, ghrelin, melatonin and glucose (Goel et al. 2009). It appears that most overweight and obese people sleep less than normal, and therefore, they have more time to eat (Chaput et al. 2011) and snack (Nedeltcheva et al. 2009b), and they are also at increased risk of insulin resistance and type 2 diabetes (Chao et al. 2011) (Fig. 2). Interestingly, sleep architecture changes with seasons, with increased rapid eye movement (REM) sleep during the winter (Kohsaka et al. 1992), though any possible link with obesity etc. is unclear.

The exact mechanism linking sleep disturbances and obesity remains unclear. It has been postulated that loss of sleep reduces glucose tolerance and increases insulin resistance (Nedeltcheva et al. 2009a); it may also decrease energy expenditure (Benedict et al. 2011) through decreased secretion of thyroid hormones (Kessler et al. 2010) and/or adiponectin (Simpson et al. 2010). On the other hand, sleep deprivation may increase food intake and appetite (Brondel et al. 2010) by decreasing leptin or by increasing ghrelin (Taheri et al. 2004) and orexin (Zeitzer 2013). Since sleep is believed to allow the brain to replenish energy stores, it has been speculated that altered sleeping habits might—through the autonomic nervous system and hypothalamic-pituitary-adrenal axis—impact on the release of metabolic hormones and the control of food intake (Spiegel et al. 2004).

### Timing and frequency of food intake

Eating patterns—when to eat, the amount of food eaten and the circumstances leading to finishing a meal—affect energy intake (Blundell and Cooling 2000). An important study on the effect of meal frequency/pattern on body fat and metabolic functions in humans was by Fábry and Tepperman (1970), who found that excessive weight, hypercholesterolemia, impaired glucose tolerance and ischemic heart disease were more common among persons who ate larger meals less frequently rather than smaller meals more often. Individuals can be classified as “grazers” or “gorgers.” Grazers, who eat smaller meals throughout the daytime, may be metabolically advantaged compared to gorgers (who eat fewer but larger meals), since larger meals may lead to increased fat synthesis and storage (Verboeket-van de Venne and Westerterp 1991).

It has been demonstrated that the same meal eaten at different times of the day may exert different metabolic effects; thus, it appears that a morning meal is associated with better control of body mass than when the same meal is eaten later in the day (Keim et al. 1997). Such an effect may be related to the amount of physical work performed during the daytime and endocrine responses to food intake (with the insulin response to food intake being time-of-day dependent, for example). In this respect, it has recently been demonstrated in mice that a feeding regimen that restricted feeding time but not calorie intake showed improved nutrient utilization and energy expenditure (Hatori et al. 2012). Whether this observation applies also to humans remains to be investigated.

Patterns of food intake can be determined by the social context, time availability and night work. “Binge eating” involves food intake greatly in excess of metabolic requirements, often with the loss of control over the amount eaten. It has been observed that breakfast was the least, and dinner the most, common meal associated with this practice, and binge eating was often associated with evening snacking (Harvey et al. 2011; Stunkard and Allison 2003).

A positive relationship between appetite and food intake no longer exists for meals eaten during the working day; in these circumstances, a proper meal is often replaced by “fast food” eaten at amounts reflecting time availability rather than appetite. By contrast, if there is plenty of time and one is with friends, food intake is often in excess of metabolic requirements, particularly if alcohol is drunk as part of the occasion’s conviviality (de Castro 2009; Waterhouse et al. 2005). The type of food eaten varies also over the day with greater intake of carbohydrates at breakfast and of fat at dinner (Westerterp-Plantenga et al. 1996). Short and irregular sleeps are associated with consuming more fats, fast foods and sweet beverages, and less vegetables (Baron et al. 2011), as well as with more palatable foods high in sugar, fat and salt rather than rich in protein or roughage (St-Onge et al. 2011). These problems are particularly evident when snacking late at night or during a nocturnal waking episode.

The mechanisms linking frequency of food intake and the type of food eaten remain unclear, but it has been suggested that lower leptin and higher ghrelin concentrations are involved (Taheri et al. 2004). For example, meals with a high fat-to-carbohydrate ratio decrease plasma concentrations of leptin (Havel et al. 1999) and ghrelin (Monteleone et al. 2003) concentrations.

### Night work

Shift and night work may disturb 24-h rhythms, including endocrine rhythms (Morris et al. 2012). Night work is associated with an increased risk of metabolic syndrome (Esquirol et al. 2009), obesity (Pandalai et al. 2013) and sleep disturbances (Ohayon et al. 2010). Night workers have altered eating habits (Waterhouse et al. 2003) and tend to snack (on foods high in salt and carbohydrate) rather than eat a full meal during the shift. In addition, attempts to eat with their families whenever possible often mean that total daily food intake increases. The combination of these factors increases the likelihood of developing indigestion, obesity and metabolic disorders (Karlsson et al. 2001). Lack of synchrony between body clocks and lifestyle may change the secretory profiles of metabolic hormones, including ghrelin and leptin (Crispim et al. 2011), which may contribute to increased appetite and higher energy intake (Spiegel et al. 2004). Gaining weight is more likely if night workers also experience social disturbances due to their abnormal lifestyle (Waterhouse et al. 2005).

### Disruption of adipose tissue rhythms

Genetic expression in adipose tissue seems to alter in obesity. Thus, disturbed expression of clock genes in WAT was detected in genetically obese mice of the KK and KK-A(y) strains (Ando et al. 2005). These mice showed also abnormal leptin secretion, sleep disturbances, altered locomotor activity and changed rhythms of adiponectin and resistin release. Also, mice with adipose tissue-targeted deletion of BMAL1 displayed increased adiposity and body weight, impairment of feeding rhythms and alterations in the expression of hypothalamic neuropeptides that regulate appetite (Paschos et al. 2012). Similarly, obese humans were found to differ in the expression patterns of several clock and metabolic genes in adipose tissue (Garaulet et al. 2011), in the rhythms of plasma adipokines (Saad et al. 1998) and in daily rhythms of leptin and ghrelin secretion (Heptulla et al. 2001; Yildiz et al. 2004). In addition to being a key component of the body clock, adipocyte BMAL1 has also been implicated in adipose tissue differentiation and lipogenesis (Shimba et al. 2005). Thus, it might play a role in the development of obesity. Moreover, the expression of uncoupling protein-1 (UCP1) that is in involved in BAT thermogenesis was found to associate with winter accumulation of visceral fat (Nakayama et al. 2013).

## Conclusions and perspectives

The central and peripheral clocks act to coordinate behavioural and metabolic responses with the environment. Light–dark cycles entrain the central clock in the SCN, which then synchronizes peripheral clocks and the rest of the body through autonomic innervation, body temperature, endocrine signalling and feeding-related cues. Feeding can regulate peripheral clocks independent of the central clock through local metabolites and signalling pathways. Increasing evidence suggests that this harmony may become disturbed either through behavioural misalignment (such as shift work or jet lag) or metabolic challenges (e.g. high-fat feeding) (Bass and Takahashi 2010). This may lead to further weakening of links between the clocks and result in abnormalities that promote weight gain, obesity and development of metabolic syndrome. Future research will need to elucidate the exact molecular mechanisms linking biological clocks with metabolic homeostasis and nutrient state. Ultimately, such studies may help to prevent metabolic derangement and obesity in individuals with sleeping disorders or working on shifts and to optimize weight loss regimens.

Also, whilst there is considerable evidence that altered daily rhythms are associated with obesity and allied problems, there is an interpretive problem associated with such results. As already mentioned, a measured daily rhythm reflects not only the activity of the central and peripheral oscillators controlling metabolism but also exogenous effects directly due to the pattern of food intake (including the period of fasting during sleep). That is, a measured rhythm is not necessarily an accurate reflection of the activities of the internal mechanisms (the oscillators); it might be “masked” by the exogenous component. It is important to distinguish between these two causes of a rhythm if detailed information regarding the interactions between the internal oscillators and metabolic processes in obesity is sought. One way to separate the effects of these two causes is to minimize the exogenous component; this could be achieved by giving identical meals at equal time intervals throughout the 24 h, also prohibiting sleep. The rhythms observed in these circumstances (when the patterns of food intake and the sleep-wake cycle have been removed) would then reflect those of the internal processes far more closely. Such studies are of fundamental importance to a better understanding of obesity and need to be performed in future research.

## References

[CR1] Adam CL, Mercer JG (2004). Appetite regulation and seasonality: implications for obesity. Proc Nutr Soc.

[CR2] Adamantidis A, de Lecea L (2008). Sleep and metabolism: shared circuits, new connections. Trends Endocrinol Metab.

[CR3] Albrecht U (2012). Timing to perfection: the biology of central and peripheral circadian clocks. Neuron.

[CR4] Ando H, Yanagihara H, Hayashi Y, Obi Y, Tsuruoka S, Takamura T, Kaneko S, Fujimura A (2005). Rhythmic messenger ribonucleic acid expression of clock genes and adipocytokines in mouse visceral adipose tissue. Endocrinology.

[CR5] Au-Yong IT, Thorn N, Ganatra R, Perkins AC, Symonds ME (2009). Brown adipose tissue and seasonal variation in humans. Diabetes.

[CR6] Balsalobre A, Brown SA, Marcacci L, Tronche F, Kellendonk C, Reichardt HM, Schutz G, Schibler U (2000). Resetting of circadian time in peripheral tissues by glucocorticoid signaling. Science.

[CR7] Baron KG, Reid KJ, Kern AS, Zee PC (2011). Role of sleep timing in caloric intake and BMI. Obesity (Silver Spring).

[CR8] Bass J (2012). Circadian topology of metabolism. Nature.

[CR9] Bass J, Takahashi JS (2010). Circadian integration of metabolism and energetics. Science.

[CR10] Benedict C, Hallschmid M, Lassen A, Mahnke C, Schultes B, Schioth HB, Born J, Lange T (2011). Acute sleep deprivation reduces energy expenditure in healthy men. Am J Clin Nutr.

[CR11] Blundell JE, Cooling J (2000). Routes to obesity: phenotypes, food choices and activity. Br J Nutr.

[CR12] Bostwick J, Nguyen D, Cornelissen G, Halberg F, Hoogerwerf WA (2010). Effects of acute and chronic STZ-induced diabetes on clock gene expression and feeding in the gastrointestinal tract. Mol Cell Biochem.

[CR13] Boulos R, Vikre EK, Oppenheimer S, Chang H, Kanarek RB (2012). ObesiTV: how television is influencing the obesity epidemic. Physiol Behav.

[CR14] Brondel L, Romer MA, Nougues PM, Touyarou P, Davenne D (2010). Acute partial sleep deprivation increases food intake in healthy men. Am J Clin Nutr.

[CR15] Chao CY, Wu JS, Yang YC, Shih CC, Wang RH, Lu FH, Chang CJ (2011). Sleep duration is a potential risk factor for newly diagnosed type 2 diabetes mellitus. Metabolism.

[CR16] Chaput JP, Sjodin AM, Astrup A, Despres JP, Bouchard C, Tremblay A (2010). Risk factors for adult overweight and obesity: the importance of looking beyond the “big two”. Obes Facts.

[CR17] Chaput JP, Despres JP, Bouchard C, Tremblay A (2011). The association between short sleep duration and weight gain is dependent on disinhibited eating behavior in adults. Sleep.

[CR18] Colles SL, Dixon JB, O’Brien PE (2007). Night eating syndrome and nocturnal snacking: association with obesity, binge eating and psychological distress. Int J Obes (Lond).

[CR19] Crispim CA, Waterhouse J, Damaso AR, Zimberg IZ, Padilha HG, Oyama LM, Tufik S, de Mello MT (2011). Hormonal appetite control is altered by shift work: a preliminary study. Metabolism.

[CR20] Cummings DE (2006). Ghrelin and the short- and long-term regulation of appetite and body weight. Physiol Behav.

[CR21] de Castro JM (1991). Seasonal rhythms of human nutrient intake and meal pattern. Physiol Behav.

[CR22] de Castro JM (2009). When, how much and what foods are eaten are related to total daily food intake. Br J Nutr.

[CR23] Dibner C, Schibler U, Albrecht U (2010). The mammalian circadian timing system: organization and coordination of central and peripheral clocks. Annu Rev Physiol.

[CR24] Ebling FJ, Barrett P (2008). The regulation of seasonal changes in food intake and body weight. J Neuroendocrinol.

[CR25] Eckel-Mahan K, Sassone-Corsi P (2013). Metabolism and the circadian clock converge. Physiol Rev.

[CR26] Ekmekcioglu C, Touitou Y (2011). Chronobiological aspects of food intake and metabolism and their relevance on energy balance and weight regulation. Obes Rev.

[CR27] Esquirol Y, Bongard V, Mabile L, Jonnier B, Soulat JM, Perret B (2009). Shift work and metabolic syndrome: respective impacts of job strain, physical activity, and dietary rhythms. Chronobiol Int.

[CR28] Fábry P, Tepperman J (1970). Meal frequency—a possible factor in human pathology. Am J Clin Nutr.

[CR29] Finucane MM, Stevens GA, Cowan MJ, Danaei G, Lin JK, Paciorek CJ, Singh GM, Gutierrez HR, Lu Y, Bahalim AN, Farzadfar F, Riley LM, Ezzati M (2011). National, regional, and global trends in body-mass index since 1980: systematic analysis of health examination surveys and epidemiological studies with 960 country-years and 9.1 million participants. Lancet.

[CR30] Florant GL, Healy JE (2012). The regulation of food intake in mammalian hibernators: a review. J Comp Physiol B.

[CR31] Funato H, Tsai AL, Willie JT, Kisanuki Y, Williams SC, Sakurai T, Yanagisawa M (2009). Enhanced orexin receptor-2 signaling prevents diet-induced obesity and improves leptin sensitivity. Cell Metab.

[CR32] Garaulet M, Ordovas JM, Gomez-Abellan P, Martinez JA, Madrid JA (2011). An approximation to the temporal order in endogenous circadian rhythms of genes implicated in human adipose tissue metabolism. J Cell Physiol.

[CR33] Gavrila A, Chan JL, Yiannakouris N, Kontogianni M, Miller LC, Orlova C, Mantzoros CS (2003). Serum adiponectin levels are inversely associated with overall and central fat distribution but are not directly regulated by acute fasting or leptin administration in humans: cross-sectional and interventional studies. J Clin Endocrinol Metab.

[CR34] Goel N, Stunkard AJ, Rogers NL, Van Dongen HP, Allison KC, O’Reardon JP, Ahima RS, Cummings DE, Heo M, Dinges DF (2009). Circadian rhythm profiles in women with night eating syndrome. J Biol Rhythm.

[CR35] Harvey K, Rosselli F, Wilson GT, Debar LL, Striegel-Moore RH (2011). Eating patterns in patients with spectrum binge-eating disorder. Int J Eat Disord.

[CR36] Hatori M, Vollmers C, Zarrinpar A, DiTacchio L, Bushong EA, Gill S, Leblanc M, Chaix A, Joens M, Fitzpatrick JA, Ellisman MH, Panda S (2012). Time-restricted feeding without reducing caloric intake prevents metabolic diseases in mice fed a high-fat diet. Cell Metab.

[CR37] Havel PJ, Townsend R, Chaump L, Teff K (1999). High-fat meals reduce 24-h circulating leptin concentrations in women. Diabetes.

[CR38] Heptulla R, Smitten A, Teague B, Tamborlane WV, Ma YZ, Caprio S (2001). Temporal patterns of circulating leptin levels in lean and obese adolescents: relationships to insulin, growth hormone, and free fatty acids rhythmicity. J Clin Endocrinol Metab.

[CR39] Hoogerwerf WA (2010). Role of clock genes in gastrointestinal motility. Am J Physiol Gastrointest Liver Physiol.

[CR40] Johnson F, Mavrogianni A, Ucci M, Vidal-Puig A, Wardle J (2011). Could increased time spent in a thermal comfort zone contribute to population increases in obesity?. Obes Rev.

[CR41] Johnston JD (2012). Adipose circadian rhythms: translating cellular and animal studies to human physiology. Mol Cell Endocrinol.

[CR42] Kanikowska D, Sato M, Sugenoya J, Shimizu Y, Nishimura N, Inukai Y, Iwase S (2013). Attenuated thermoregulatory responses with increased plasma osmolality in obese subjects during two seasons. Int J Biometeorol.

[CR43] Karlsson B, Knutsson A, Lindahl B (2001). Is there an association between shift work and having a metabolic syndrome? Results from a population based study of 27,485 people. Occup Environ Med.

[CR44] Keim NL, Van Loan MD, Horn WF, Barbieri TF, Mayclin PL (1997). Weight loss is greater with consumption of large morning meals and fat-free mass is preserved with large evening meals in women on a controlled weight reduction regimen. J Nutr.

[CR45] Kessler L, Nedeltcheva A, Imperial J, Penev PD (2010). Changes in serum TSH and free T4 during human sleep restriction. Sleep.

[CR46] Klein S, Burke LE, Bray GA, Blair S, Allison DB, Pi-Sunyer X, Hong Y, Eckel RH (2004). Clinical implications of obesity with specific focus on cardiovascular disease: a statement for professionals from the American Heart Association Council on Nutrition, Physical Activity, and Metabolism: endorsed by the American College of Cardiology Foundation. Circulation.

[CR47] Kohsaka M, Fukuda N, Honma K, Honma S, Morita N (1992). Seasonality in human sleep. Experientia.

[CR48] Kotagal S, Krahn LE, Slocumb N (2004). A putative link between childhood narcolepsy and obesity. Sleep Med.

[CR49] Kriegsfeld LJ, Silver R (2006). The regulation of neuroendocrine function: timing is everything. Horm Behav.

[CR50] Little TJ, Horowitz M, Feinle-Bisset C (2005). Role of cholecystokinin in appetite control and body weight regulation. Obes Rev.

[CR51] Liu C, Li S, Liu T, Borjigin J, Lin JD (2007). Transcriptional coactivator PGC-1alpha integrates the mammalian clock and energy metabolism. Nature.

[CR52] Lowden A, Moreno C, Holmback U, Lennernas M, Tucker P (2010). Eating and shift work—effects on habits, metabolism and performance. Scand J Work Environ Health.

[CR53] Lumeng CN, Saltiel AR (2011). Inflammatory links between obesity and metabolic disease. J Clin Invest.

[CR54] Marcheva B, Ramsey KM, Buhr ED, Kobayashi Y, Su H, Ko CH, Ivanova G, Omura C, Mo S, Vitaterna MH, Lopez JP, Philipson LH, Bradfield CA, Crosby SD, JeBailey L, Wang X, Takahashi JS, Bass J (2010). Disruption of the clock components CLOCK and BMAL1 leads to hypoinsulinaemia and diabetes. Nature.

[CR55] Marcheva B, Ramsey KM, Peek CB, Affinati A, Maury E, Bass J (2013) Circadian clocks and metabolism. Handb Exp Pharmacol 127–155 10.1007/978-3-642-25950-0_6 PMC4089089 23604478

[CR56] Mieda M, Williams SC, Richardson JA, Tanaka K, Yanagisawa M (2006). The dorsomedial hypothalamic nucleus as a putative food-entrainable circadian pacemaker. Proc Natl Acad Sci U S A.

[CR57] Mistlberger RE (2011). Neurobiology of food anticipatory circadian rhythms. Physiol Behav.

[CR58] Mohawk JA, Green CB, Takahashi JS (2012). Central and peripheral circadian clocks in mammals. Annu Rev Neurosci.

[CR59] Monteleone P, Bencivenga R, Longobardi N, Serritella C, Maj M (2003). Differential responses of circulating ghrelin to high-fat or high-carbohydrate meal in healthy women. J Clin Endocrinol Metab.

[CR60] Morris CJ, Aeschbach D, Scheer FA (2012). Circadian system, sleep and endocrinology. Mol Cell Endocrinol.

[CR61] Mullington JM, Chan JL, Van Dongen HP, Szuba MP, Samaras J, Price NJ, Meier-Ewert HK, Dinges DF, Mantzoros CS (2003). Sleep loss reduces diurnal rhythm amplitude of leptin in healthy men. J Neuroendocrinol.

[CR62] Nakayama K, Miyashita H, Yanagisawa Y, Iwamoto S (2013). Seasonal effects of UCP1 gene polymorphism on visceral fat accumulation in Japanese adults. PLoS ONE.

[CR63] Naslund E, Hellstrom PM (2007). Appetite signaling: from gut peptides and enteric nerves to brain. Physiol Behav.

[CR64] Nedeltcheva AV, Kessler L, Imperial J, Penev PD (2009). Exposure to recurrent sleep restriction in the setting of high caloric intake and physical inactivity results in increased insulin resistance and reduced glucose tolerance. J Clin Endocrinol Metab.

[CR65] Nedeltcheva AV, Kilkus JM, Imperial J, Kasza K, Schoeller DA, Penev PD (2009). Sleep curtailment is accompanied by increased intake of calories from snacks. Am J Clin Nutr.

[CR66] Nojkov B, Rubenstein JH, Chey WD, Hoogerwerf WA (2010). The impact of rotating shift work on the prevalence of irritable bowel syndrome in nurses. Am J Gastroenterol.

[CR67] Ohayon MM, Smolensky MH, Roth T (2010). Consequences of shiftworking on sleep duration, sleepiness, and sleep attacks. Chronobiol Int.

[CR68] Pandalai SP, Schulte PA, Miller DB (2013). Conceptual heuristic models of the interrelationships between obesity and the occupational environment. Scand J Work Environ Health.

[CR69] Pappa KI, Gazouli M, Anastasiou E, Iliodromiti Z, Antsaklis A, Anagnou NP (2013). Circadian clock gene expression is impaired in gestational diabetes mellitus. Gynecol Endocrinol.

[CR70] Paschos GK, Ibrahim S, Song WL, Kunieda T, Grant G, Reyes TM, Bradfield CA, Vaughan CH, Eiden M, Masoodi M, Griffin JL, Wang F, Lawson JA, Fitzgerald GA (2012). Obesity in mice with adipocyte-specific deletion of clock component Arntl. Nat Med.

[CR71] Plasqui G, Westerterp KR (2004). Seasonal variation in total energy expenditure and physical activity in Dutch young adults. Obes Res.

[CR72] Reilly T, Peiser B (2006). Seasonal variations in health-related human physical activity. Sports Med.

[CR73] Reinberg A (1972). The significance of biological rhythms in biometeorology. Biological rhythms and human biometeorology, with special reference to mortality rhythms and chronotoxicology. Int J Biometeorol.

[CR74] Saad MF, Riad-Gabriel MG, Khan A, Sharma A, Michael R, Jinagouda SD, Boyadjian R, Steil GM (1998). Diurnal and ultradian rhythmicity of plasma leptin: effects of gender and adiposity. J Clin Endocrinol Metab.

[CR75] Sadacca LA, Lamia KA, deLemos AS, Blum B, Weitz CJ (2011). An intrinsic circadian clock of the pancreas is required for normal insulin release and glucose homeostasis in mice. Diabetologia.

[CR76] Saito M, Okamatsu-Ogura Y, Matsushita M, Watanabe K, Yoneshiro T, Nio-Kobayashi J, Iwanaga T, Miyagawa M, Kameya T, Nakada K, Kawai Y, Tsujisaki M (2009). High incidence of metabolically active brown adipose tissue in healthy adult humans: effects of cold exposure and adiposity. Diabetes.

[CR77] Sakurai T, Amemiya A, Ishii M, Matsuzaki I, Chemelli RM, Tanaka H, Williams SC, Richardson JA, Kozlowski GP, Wilson S, Arch JR, Buckingham RE, Haynes AC, Carr SA, Annan RS, McNulty DE, Liu WS, Terrett JA, Elshourbagy NA, Bergsma DJ, Yanagisawa M (1998). Orexins and orexin receptors: a family of hypothalamic neuropeptides and G protein-coupled receptors that regulate feeding behavior. Cell.

[CR78] Shimba S, Ishii N, Ohta Y, Ohno T, Watabe Y, Hayashi M, Wada T, Aoyagi T, Tezuka M (2005). Brain and muscle Arnt-like protein-1 (BMAL1), a component of the molecular clock, regulates adipogenesis. Proc Natl Acad Sci U S A.

[CR79] Simpson NS, Banks S, Arroyo S, Dinges DF (2010). Effects of sleep restriction on adiponectin levels in healthy men and women. Physiol Behav.

[CR80] Solt LA, Wang Y, Banerjee S, Hughes T, Kojetin DJ, Lundasen T, Shin Y, Liu J, Cameron MD, Noel R, Yoo SH, Takahashi JS, Butler AA, Kamenecka TM, Burris TP (2012). Regulation of circadian behaviour and metabolism by synthetic REV-ERB agonists. Nature.

[CR81] Spiegel K, Leproult R, L’hermite-Baleriaux M, Copinschi G, Penev PD, Van CE (2004). Leptin levels are dependent on sleep duration: relationships with sympathovagal balance, carbohydrate regulation, cortisol, and thyrotropin. J Clin Endocrinol Metab.

[CR82] Stephan FK (2002). The “other” circadian system: food as a Zeitgeber. J Biol Rhythm.

[CR83] St-Onge MP, Roberts AL, Chen J, Kelleman M, O’Keeffe M, RoyChoudhury A, Jones PJ (2011). Short sleep duration increases energy intakes but does not change energy expenditure in normal-weight individuals. Am J Clin Nutr.

[CR84] Stunkard AJ, Allison KC (2003). Two forms of disordered eating in obesity: binge eating and night eating. J Obes Relat Metab Disord.

[CR85] Stutz AM, Staszkiewicz J, Ptitsyn A, Argyropoulos G (2007). Circadian expression of genes regulating food intake. Obesity (Silver Spring).

[CR86] Suzuki K, Jayasena CN, Bloom SR (2012). Obesity and appetite control. Exp Diabetes Res.

[CR87] Taheri S, Lin L, Austin D, Young T, Mignot E (2004). Short sleep duration is associated with reduced leptin, elevated ghrelin, and increased body mass index. PLoS Med.

[CR88] Tsujino N, Sakurai T (2013). Role of orexin in modulating arousal, feeding, and motivation. Front Behav Neurosci.

[CR89] van der Veen DR, Shao J, Chapman S, Leevy WM, Duffield GE (2012). A diurnal rhythm in glucose uptake in brown adipose tissue revealed by in vivo PET-FDG imaging. Obesity (Silver Spring).

[CR90] Verboeket-van de Venne WP, Westerterp KR (1991). Influence of the feeding frequency on nutrient utilization in man: consequences for energy metabolism. Eur J Clin Nutr.

[CR91] Waterhouse J, Buckley P, Edwards B, Reilly T (2003). Measurement of, and some reasons for, differences in eating habits between night and day workers. Chronobiol Int.

[CR92] Waterhouse J, Bailey L, Tomlinson F, Edwards B, Atkinson G, Reilly T (2005). Food intake in healthy young adults: effects of time pressure and social factors. Chronobiol Int.

[CR93] Welsh DK, Takahashi JS, Kay SA (2010). Suprachiasmatic nucleus: cell autonomy and network properties. Annu Rev Physiol.

[CR94] Westerterp-Plantenga MS, Ijedema MJ, Wijckmans-Duijsens NE (1996). The role of macronutrient selection in determining patterns of food intake in obese and non-obese women. Eur J Clin Nutr.

[CR95] Wyse CA, Selman C, Page MM, Coogan AN, Hazlerigg DG (2011). Circadian desynchrony and metabolic dysfunction; did light pollution make us fat?. Med Hypotheses.

[CR96] Yildiz BO, Suchard MA, Wong ML, McCann SM, Licinio J (2004). Alterations in the dynamics of circulating ghrelin, adiponectin, and leptin in human obesity. Proc Natl Acad Sci U S A.

[CR97] Zeitzer JM (2013). Control of sleep and wakefulness in health and disease. Prog Mol Biol Transl Sci.

[CR98] Zvonic S, Ptitsyn AA, Conrad SA, Scott LK, Floyd ZE, Kilroy G, Wu X, Goh BC, Mynatt RL, Gimble JM (2006). Characterization of peripheral circadian clocks in adipose tissues. Diabetes.

